# Tetracycline Adsorption on Magnetic Sludge Biochar: Effects of pH, Humic Acid (HA), and Fulvic Acid (FA)

**DOI:** 10.3390/mi13071057

**Published:** 2022-06-30

**Authors:** Yuanhui Wu, Meizhi Yang, Dan Long, Fanian Yang, Suxing Luo

**Affiliations:** 1 Department of Chemistry and Chemical Engineering, Zunyi Normal University, Zunyi 563006, China; ddanlong@163.com (D.L.); fanyangn@126.com (F.Y.); 2 Special Key Laboratory of Electrochemistry for Materials of Guizhou Province, Zunyi 563006, China; 3 Office of Academic Research, Guizhou Open University, Guiyang 550023, China; yangmeizhi@mail.gyig.ac.cn

**Keywords:** adsorption, fulvic acid, humic acid, magnetic sludge biochar, tetracycline

## Abstract

Natural organic matters (NOMs) are ubiquitous in the environment, but few systematic studies have examined the influence of NOMs on the sorption ability of magnetic sludge biochar. In this study, magnetic sludge biochar was synthesized, characterized, and used as a sorbent to remove tetracycline (TC) from aqueous solutions. The effects of pH, humic acid (HA), and fulvic acid (FA) on TC adsorption by magnetic sludge biochar were studied using batch experiments. Adding HA and FA can alter the adsorption behavior of TC, except for its pH dependency. The results of this study show that relatively low concentrations of dissolved HA (≤8 ppm) and FA (≤5 ppm) promote the adsorption capacity of TC, but higher concentrations compete against TC for sorption sites on the surface of magnetic sludge biochar. The results of this study promote a better understanding of the application of magnetic sludge biochar in real antibiotic wastewater.

## 1. Introduction

Magnetic biochar is commonly prepared by impregnation–pyrolysis, chemical co-precipitation, solvothermal, and reductive co-precipitation [1,2,3]. Magnetic biochar has a high surface area, porous structure, appreciable amounts of active sites on its surface, and good recyclability [4]. Furthermore, using excess sludge to prepare magnetic sludge biochar could reduce its preparation cost and have great significance in solving the secondary pollution of inadequately treated sludge [5]. Owing to its extraordinary adsorption capacity, magnetic sludge biochar has attracted much attention in terms of the removal of various pollutants, including antibiotics, heavy metals, and dyes [6,7,8].

Humic acid (HA) and fulvic acid (FA), as two important parts of dissolved organic matter, are ubiquitous in almost all aquatic ecosystems at concentrations typically ranging from 0.1 to 10 ppm [9]. The abundant functional groups (aliphatic and aromatic COOH, OH, OCH_3_, and aliphatic CO) and special physicochemical properties (hydrophobicity, aromaticity, and functionality) [10] could give HA and FA a strong affinity for biochar. Therefore, HA and FA are generally chosen as model NOM molecules in sorption studies. As reported previously, HA and FA could participate in the interaction between biochar and pollutants [11]. They could also modify the surface properties of biochar and affect its adsorption behavior towards various pollutants. Moreover, they could also result in site competition and pore blockage [12]. Lian et al. [13] reported that HA-coated biochar (prepared by straws) enhanced the adsorption of sulfamethoxazole but suppressed that of sulfanilamide on biochar. The effects of adsorbed HA on sulfonamide sorption by biochar were related to the properties of sorbate, the composition and structure of the HA adlayer, and concentration. Luo et al. [12] demonstrated that low concentrations of dissolved HA promoted the removal of ciprofloxacin by sludge biochar, but higher concentrations of dissolved HA suppressed ciprofloxacin removal. Park et al. [14] indicated that HA could change the charge of biochar and increase sorption sites resulting from less aggregation of biochar particles. In conclusion, the effect of NOM on the adsorption behavior of biochar varied greatly, which mainly depended on the properties of the biochar, sorbents, NOM types, and NOM concentrations. However, very little attention has been paid to the effects of NOM on the sorption of pollutants by magnetic sludge biochar, especially as a function of pH. 

The extensive use of antibiotics has become a serious environmental problem, which could discharge into various media [15,16] and cause adverse consequences [16]. Among the commonly used antibiotics, tetracycline (TC) is the most widely used in aquaculture and pharmacy due to its relatively low cost [17]. TC has been detected in wastewater, surface, and groundwater, and might cause harm to human health and ecosystems [18,19]. Therefore, in this study, TC was selected as the target organic pollutant.

The objective of this study was to investigate the sorption behavior of TC on magnetic sludge biochar as a function of pH in the presence of HA and FA. The results of this study are expected to provide new insights into understanding how magnetic sludge biochar interacts with organic contaminants in a real water environment.

## 2. Materials and Methods

### 2.1. Materials

The activated sludge was sampled from Gaoqiao Wastewater Treatment Plant in Zunyi, China. Tetracycline (TC, ≥98%), ferrous chloride, and ferric chloride (≥99.9%) were purchased from Aladdin Industrial Corporation (Los Angeles, CA, USA). HA and FA were purchased from Macklin Biochemical Technology and Wengjiang Reagent Co., Ltd. (Shaoguan, China). All other chemicals were of analytical grade and used without any further purification.

### 2.2. Preparation of Magnetic Sludge Biochar 

Firstly, 5 mL of 1 mol/L HCl was added to 500 mL of sludge (suspended solid concentration: 14.0 g/L). In order to destroy the cell wall, the agitated mixture (400 rpm, 20 min) was transferred to an ultrasonic cell crusher, pretreated under 59 kHz for 15 min, and then dried at 105 °C overnight. Secondly, magnetic sludge biochar was prepared according to the reference [20]. For forming Fe_3_O_4_ particles on the sludge biochar surface, 5.28 g of FeCl_2_ and 13.50 g of FeCl_3_ were dissolved in 500 mL of water, and then 50 g of dried sludge was added into the aqueous solution. Subsequently, 2 mol/L of NaOH solution was added to the above mixture until the pH of the suspension reached 10.0 under vigorous stirring conditions. The solid phase was separated by centrifugation at 4000 rpm before being vacuum dried at 70 °C. Finally, the composite was pyrolyzed at 400 °C for 4 h in a tubal furnace under an N_2_ atmosphere to prepare magnetic sludge biochar.

### 2.3. Characterization of Magnetic Sludge Biochar

The morphology of magnetic sludge biochar was characterized by a scanning electron microscope (SEM, Scios, FEI Company, Hillsboro, OR, USA). The specific surface area and pore size of magnetic sludge biochar were determined using BSD-PS (Beishide Instruments, Beijing, China). The surface functional groups of magnetic sludge biochar were obtained with a FTIR analyzer (VERTEX70 spectrometer, Bruker Co., Bremen, Germany). The zeta potentials of magnetic sludge biochar were determined using Malvern Zetasizer (Nano ZS90, Great Malvern, UK). The iron weight ratio in the magnetic biochar was measured by atomic absorption spectroscopy (AAS, 990SUPER, Beijing Purkinje General Instrument Co., Ltd., Beijing, China).

### 2.4. Batch Sorption Experiment

To examine the influence of HA and FA on magnetic sludge biochar adsorption of TC, the sorption experiments were conducted in the dark by adding 0.07 g of magnetic sludge biochar samples into 100 mL of 200 mg/L TC solution at room temperature/25 °C (the final dissolved concentrations of HA and FA were 0, 5, 8, 12, and 15 ppm), before being agitated in the incubator shaker at 150 rpm. To investigate the effect of pH on the adsorption behavior of TC in the above conditions, the initial pH of the TC solution was adjusted in the range of 2.0–8.0 with 0.2 mol/L of HCl or NaOH. At certain time intervals, 2.0 mL of the suspension was sampled and separated magnetically. Then, the concentrations of TC and HA in the supernatant were determined by UV–vis (UV–vis, CARY 300, Agilent, Santa Clara, CA, USA) at 357 nm and 254 nm, respectively. The concentration of FA was measured with a total organic carbon (TOC) analyzer (Shimadzu 3201, Kyoto, Japan).

## 3. Results and Discussion

### 3.1. Characterization of Magnetic Sludge Biochar

**Figure 1:**
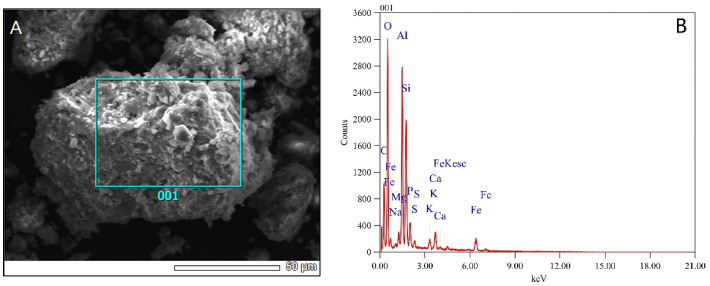
SEM image (**A**) and EDX elemental analysis (**B**) of as-prepared biochar.

The morphology of magnetic sludge biochar was characterized by SEM (Figure 1A). The surface morphology of the magnetic biochar is similar to that of activated carbon, with a large number of irregular Fe_3_O_4_ particles distributed on its surface. The SEM-EDX results (Figure 1B) confirmed the existence of Fe species. Moreover, the atom and weight ratio of iron on the surface of magnetic sludge biochar was 2.8% and 7.1%, respectively. Meanwhile, the iron weight ratio in magnetic sludge biochar bulk calculated by AAS analysis was 7.8%, which was consistent with the EDX results. The above results indicate that Fe_3_O_4_ exists in both the surface and the interior of the sludge biochar.

**Figure 2:**
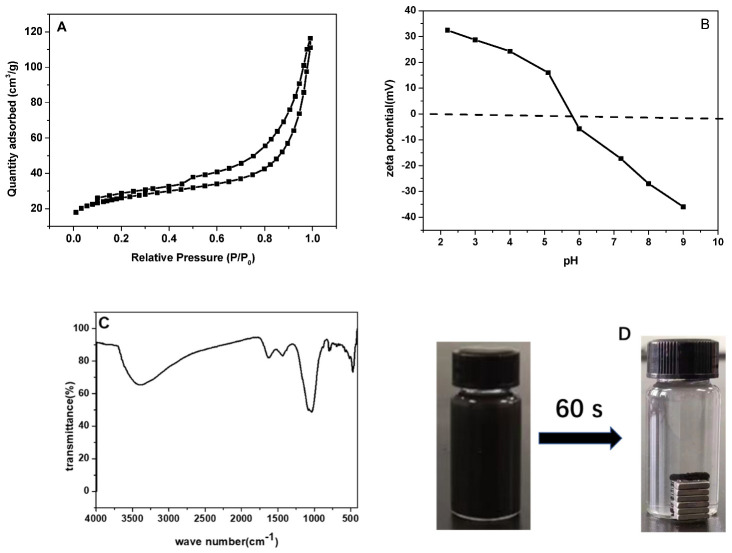
Characterizations of the magnetic biochar (**A**) N_2_ adsorption–desorption isotherm, (**B**) Zeta potential, (**C**) FT−IR, and (**D**) magnetic separation property.

The surface and pore features of as-prepared magnetic biochar were detected by a nitrogen test. As depicted in Figure 2A, the isotherm curves are consistent with the IUPAC classification type IV curve and the H_3_-type hysteresis loop. The S_BET_ was calculated as 93.41 m^2^/g, and the average pore size was 9.017 nm. The isotherm has no obvious inflection point in the low relative pressure region, indicating that the magnetic biochar has a low proportion of micropores. The lack of a saturated adsorption platform in the medium relative pressure area indicates that the biochar has irregular pores and morphologies.

Figure 2B exhibited the surface zeta potentials of magnetic sludge biochar. The isoelectric point (pH_IEP_) of magnetic biochar was estimated to be 5.7. When pH < 5.7, the surface of magnetic sludge biochar carried a positive charge, while the surface displayed a negative charge when pH > 5.7.

In order to reveal the functional groups of the as-prepared magnetic biochar, FTIR analysis was performed (Figure 2C). The strong adsorption peaks at 1037 cm^−1^ and 1633 cm^−1^ corresponded to the C-O-C stretching vibration band and C=C bonds in the aromatic compounds, respectively. The peak at 3389 cm^−1^ indicated that the magnetic biochar possessed a significant amount of -OH groups on its surface [21]. The peak at 470 cm^−1^ was ascribed to the Fe-O stretching bond [22,23]. Finally, the magnetic separation property was investigated using a separation test (Figure 2D), and the results showed that biochar could be completely separated from the TC and MS suspensions within 60 s of application of the magnets.

### 3.2. Adsorption of TC on Magnetic Sludge Biochar in the Absence of NOM

#### 3.2.1. Effect of Initial pH of TC

**Figure 3:**
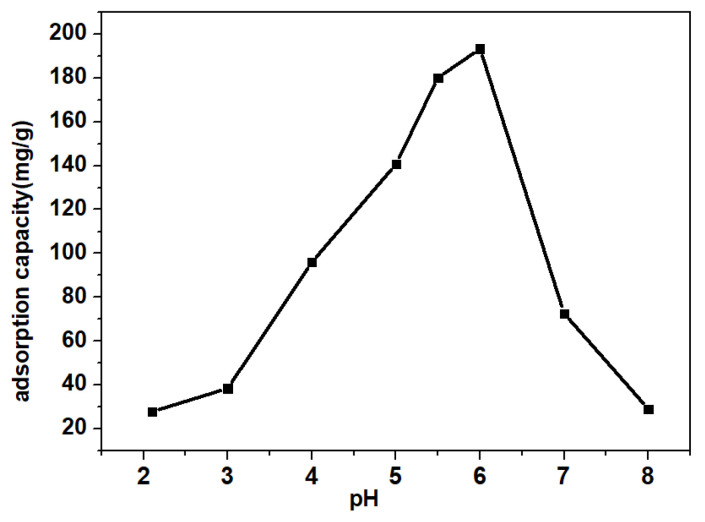
Effect of initial pH on magnetic biochar (adsorption time: 18 h).

Figure 3 exhibited that the sorption capacity of TC was markedly raised as the initial solution pH increased, and the maximum adsorption amount occurred at pH = 6. In aqueous solutions, TC will undergo protonation–deprotonation reactions and present different ionic species, including cation (TC^+^), molecule (TC°), and anions (TC^−^ and TC^2−^) as the dissociation constants (pKa) of TC are 3.3, 7.7, and 9.7 (shown in Figure S1) [5]. Apparently, when pH ≤ 3.0, the adsorption capacity of TC was very limited due to both surfaces of the prepared magnetic biochar and TC being positively charged [24]. As the pH increased, the adsorption capacity sharply increased, and the adsorption performance of TC on the magnetic biochar was maximized at pH = 6 (193.3 mg/g). Although the zeta potential of the magnetic biochar was minimal and TC mainly became neutral, the maximum adsorption capacity could be explained by the π-π electron donor–acceptor and hydrogen bonding interactions [25]. Whereas, when the pH increased beyond 6, both magnetic sludge biochar and the TC (TC^−^ and TC^2−^) were negatively charged, leading to a reduction in adsorption levels.

#### 3.2.2. Effect of Magnetic Biochar Dose 

**Figure 4:**
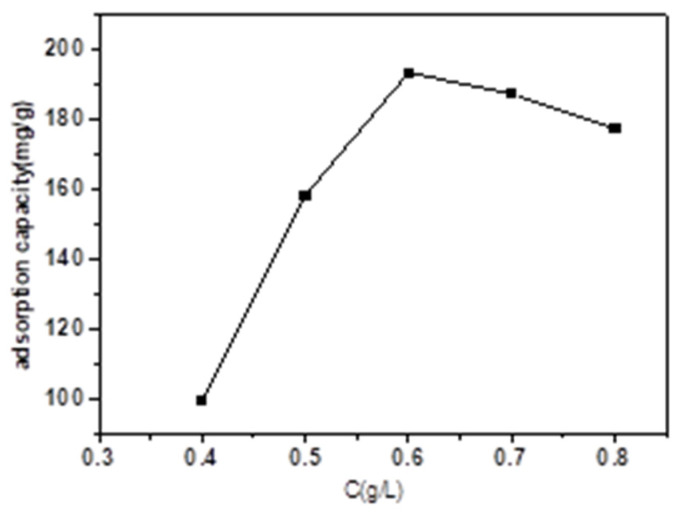
Effect of magnetic biochar dose (adsorption time: 18 h).

It was obvious that the adsorption capacity of TC increased with the magnetic biochar dose (Figure 4). A higher dose of magnetic biochar resulted in a higher adsorption capacity of TC due to more available active sites. Specifically, the adsorption capacity of TC reached the maximum when the dosage of the adsorbent was 0.6 g·L^−1^, and then decreased following further additions.

### 3.3. Effect of HA and FA on the Adsorption Behavior of TC

**Figure 5:**
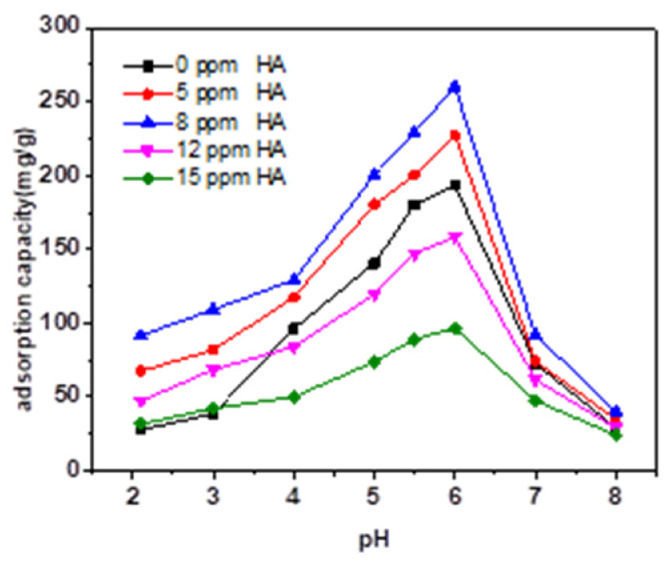
Effect of HA in different concentrations on TC adsorption by magnetic sludge biochar (adsorption time: 18 h).

**Figure 6:**
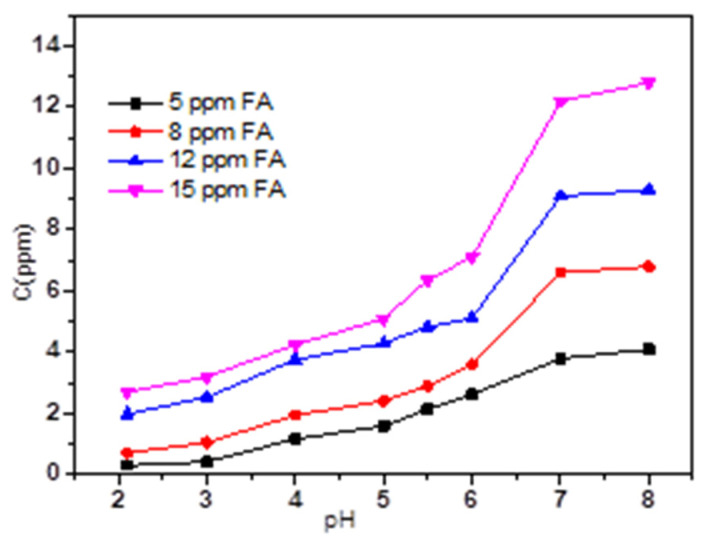
Effect of FA in different concentrations on TC adsorption by magnetic sludge biochar (adsorption time: 18 h).

**Figure 7:**
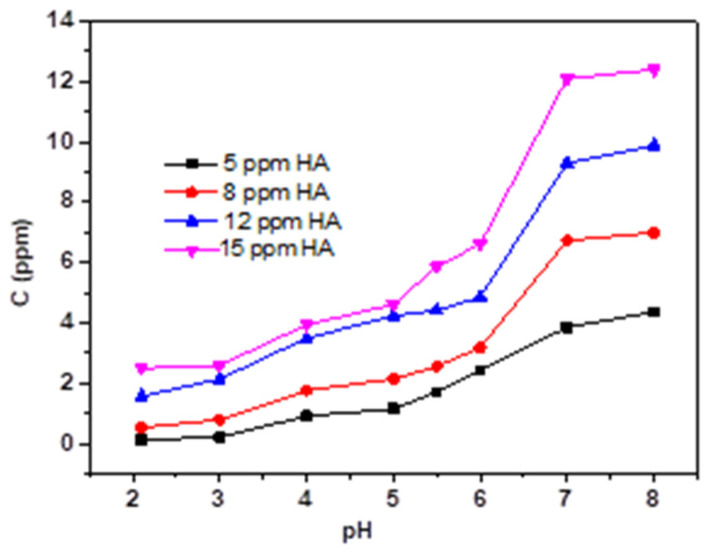
The HA concentration at the end of the experiment as a function of pH.

**Figure 8:**
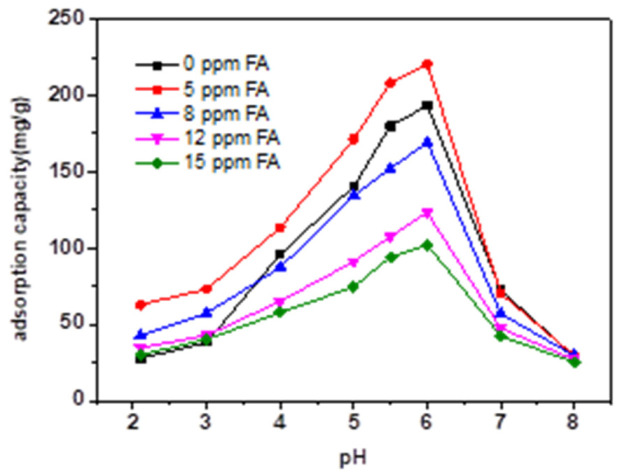
The FA concentration at the end of the experiment as a function of pH.

Figure 5 and Figure 6 present the effect of different concentrations of HA and FA on TC adsorption by magnetic sludge biochar. Overall, adding HA or FA to magnetic sludge biochar varied the adsorption behavior of TC significantly but hardly affected its global regularity of pH dependency. HA and FA contain different functional groups, including carboxylic, phenolic, and aromatic groups [26], which make them carry a negative charge over the whole pH range [27]. Therefore, HA and FA could be adsorbed onto the surface of magnetic sludge biochar. This may change its surface properties or result in competition for the surface sorption sites, thereby affecting the adsorption behavior of TC. As shown in Figure 7 and Figure 8, dissolved HA and FA concentrations at the end of the experiment partly reduced, which indicated that dissolved HA and FA were partially adsorbed onto magnetic sludge biochar over the pH range from 2.0 to 8.0. However, a simple electrostatic interaction mechanism could not completely account for the pH dependence of the sorption because when pH > pH_PZC_ (5.7), the HA, FA, and magnetic sludge biochar were all negatively charged, but as shown in Figure 6 and Figure 8, partial adsorption still occurred. Therefore, it was suspected that the functional groups of HA and FA interacted with the components of magnetic sludge biochar, which was responsible for the adsorption of HA and FA.

A low concentration of dissolved HA (8 ppm) promoted the sorption capacity of TC from 193.3 to 260.1 mg/g, which then dramatically decreased to 95.4 mg/g with further increases in dissolved HA to 15 ppm. This could be caused by competition for surface sorption sites at higher dissolved HA concentrations. Compared with the HA samples, FA has a less significant influence on the adsorption behavior of TC. Specifically, the presence of FA (5 ppm) enhanced the sorption capacity of TC from 193.3 to 220.6 mg/g, while a higher concentration of dissolved FA inhibited the TC sorption capacity. Moreover, HA and FA showed a major effect on the adsorption of TC at pH ≤ 6 and relatively little effect at higher pH values. When pH ≤ 3, the adsorption capacity of TC increased dramatically in the presence of HA and FA. This could be attributed to HA and FA adsorbed on the surface of magnetic sludge biochar, which could change its surface properties and decrease the repulsive force. As the pH increased, the functional groups of the adsorbed HA and FA could enhance the π-π electron donor–acceptor and hydrogen bonding interactions. As a result, the sorption capacities of TC were enhanced. When the pH was above 6, the dominant form of tetracycline was the anion; the HA, FA, and magnetic sludge biochar were more negatively charged; and the amount of HA and FA adsorbed on magnetic sludge biochar decreased, which resulted in a minimal effect of HA and FA on the TC adsorption process.

To further investigate the competing role of HA and FA with or without TC, the adsorption behaviors of HA and FA in the absence of TC on magnetic sludge biochar were performed, and the results are shown in Figure S2. Compared with the concentration of the residual HA and FA in the presence of TC (Figure 7 and Figure 8), the adsorption capacity of HA and FA on magnetic sludge biochar is almost unchanged with relatively low concentrations (HA ≤ 8 ppm, FA ≤ 5 ppm) and when the pH < 3.0 or pH > 6.0. Nevertheless, the adsorption capacity of HA and FA decreased significantly at pH 3–6 with higher concentrations. These results (Figure 7, Figure 8 and Figure S2) support the idea that higher concentrations of dissolved HA and FA at pH 3–6, mainly leading to competition for the surface sorption sites. The adsorbed HA and FA could, on the one hand, occupy active sites on the surface of magnetic sludge biochar but, on the other hand, repel the approaching TC molecules sterically, thus exhibiting competing behavior against the sorption of TC.

**Figure 9:**
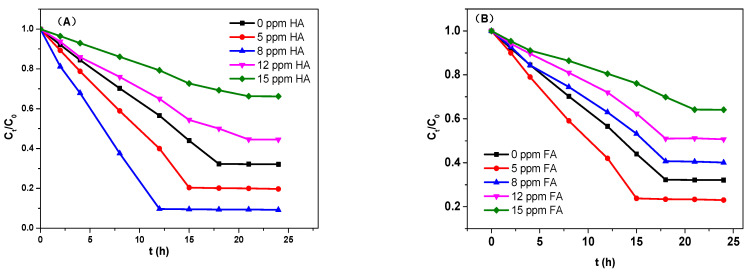
TC sorption behavior on magnetic sludge biochar as a function of the concentration of HA (**A**) and FA (**B**).

HA and FA could efficaciously affect the adsorption capacity as well as the adsorptive speed of TC. As shown in Figure 9, when the initial HA concentration was below 8 ppm, the adsorption speed was improved, and the adsorption equilibrium was achieved at 12 h. Figure 9 also showed that the dissolved HA increased to 15 ppm, the adsorption speed decreased, and the adsorption equilibrium was achieved at 18 h. Compared with the HA samples, FA affected the adsorption speed of TC less significantly, but the influence trend was similar to HA.

### 3.4. Adsorption Performance of TC on Magnetic Sludge Biochar in Coexistence of HA and FA System

Considering the co-existence of HA and FA in some actual water bodies, the adsorption performance of TC in a coexistence system was also conducted. As shown in Figure S3, the adsorption capacity and adsorptive speed were intermediate when HA and FA coexisted. Remarkably, the adsorptive equilibrium had been retained.

The effects of raw materials; biochar, NOM, and adsorbate preparation methods; and NOM on the adsorption behavior are summarized in Table 1. These findings suggest that the effects of dissolved NOM (model: HA and FA) on the adsorption of organic contaminants by biochar were significantly dependent on the properties of sorbate and biochar, as well as the types and concentration of NOM.

## 4. Conclusions

In this research, magnetic sludge biochar was successfully synthesized as an adsorbent for TC adsorption. Considering the complex influencing factors in natural systems, the adsorption behavior of TC in the absence or presence of HA and FA was investigated. The adsorption of TC on magnetic sludge biochar was greatly enhanced in the presence of low concentration HA and FA, but a higher concentration of dissolved HA and FA inhibited TC adsorption. Since the concentrations of natural organic matter in natural systems rise to a relatively high level, ranging from 0.1 to 10 ppm, magnetic sludge biochar still exhibited tremendous potential as an adsorbent for treating antibiotics in wastewater. Moreover, the application of magnetic sludge biochar for removing antibiotics can provide the dual benefits of solid waste management and wastewater treatment.

## Figures and Tables

**Table 1:** The effect of NOM on the adsorption behavior of organic contaminants by biochar.

Raw Materials	Preparation Method	NOM	Adsorbate	Effect	Reference
crop straws	pyrolysis	HA	Sulfamethoxazole sulfonamide	Declined (2.5–30 ppm) Improved (>30 ppm) Improved (2.5–30 ppm)	[13]
				declined	
				(>30 ppm)	
pine needles wheat straw sludge	pyrolysis	HA HA	polychlorinated biphenyls ciprofloxacin	Enhanced (10 ppm) Promoted (10 ppm)	[28] [12]
sludge	pyrolysis	HA FA	tetracycline	Improved (≤8 ppm) Improved (≤5 ppm)	This work

## Data Availability

All relevant data are included in the paper or its Supplementary Information.

## Supplementary Materials

The following supporting information can be downloaded at: https://www.mdpi.com/article/10.3390/mi13071057/s1, Figure S1: Various structures of TC under different conditions. Figure S2: The adsorption behaviors of HA and FA on magnetic sludge biochar as a function of pH in the absence of TC (A: HA; B: FA). Figure S3: TC sorption behavior on magnetic sludge biochar as a function of the co-existence of HA and FA.
