# Supplementary material for: Tetracycline Adsorption on Magnetic Sludge Biochar: Effects of pH, Humic Acid (HA), and Fulvic Acid (FA)
Source: Micromachines (Basel). 2022 Jun 30;13(7):1057. doi: 10.3390/mi13071057 (PMC9318179; doi:10.3390/mi13071057)
Supplement: Supplementary file 1 [file micromachines-13-01057-s001.zip › micromachines-1763837-supplementary.pdf]

micromachines

Supplementary Materials: Tetracycline Adsorption on Magnetic Sludge Biochar: Effects of pH, Humic Acid (HA), and Fulvic Acid (FA)

Yuanhui Wu [1,2,*], Meizhi Yang [3], Dan Long [1,2], Fanian Yang [1,2] and Suxing Luo [1,2,*]

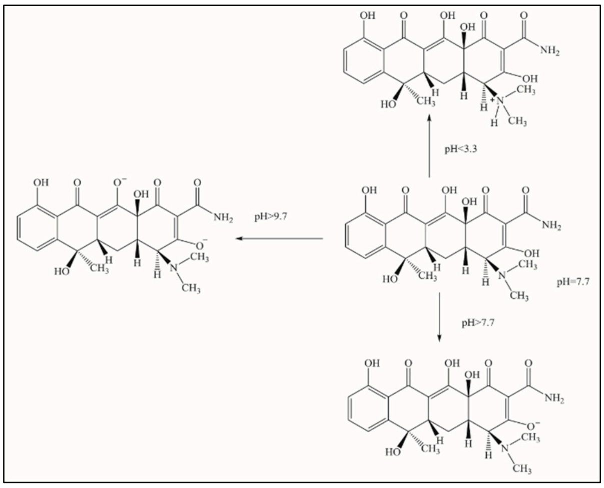

Figure S1. Various structures of TC under different conditions.

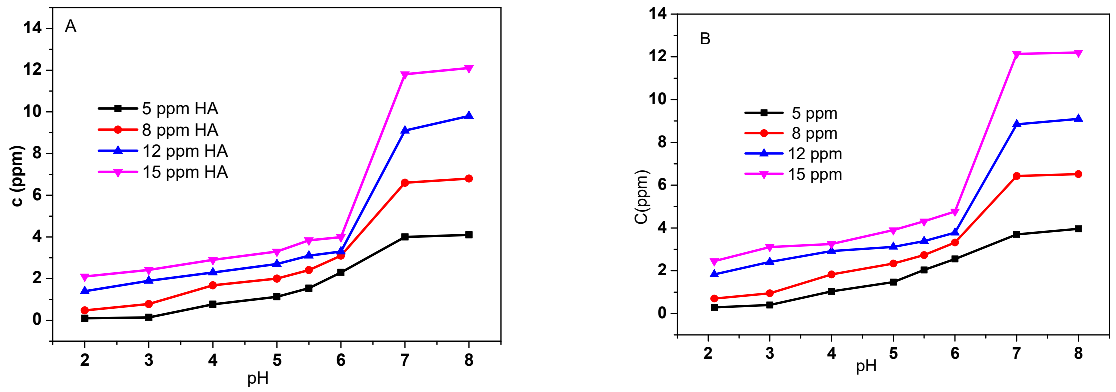

Figure S2. The adsorption behaviors of HA and FA on magnetic sludge biochar as a function of pH in the absence of TC (A: HA; B: FA).

micromachines

MDPI

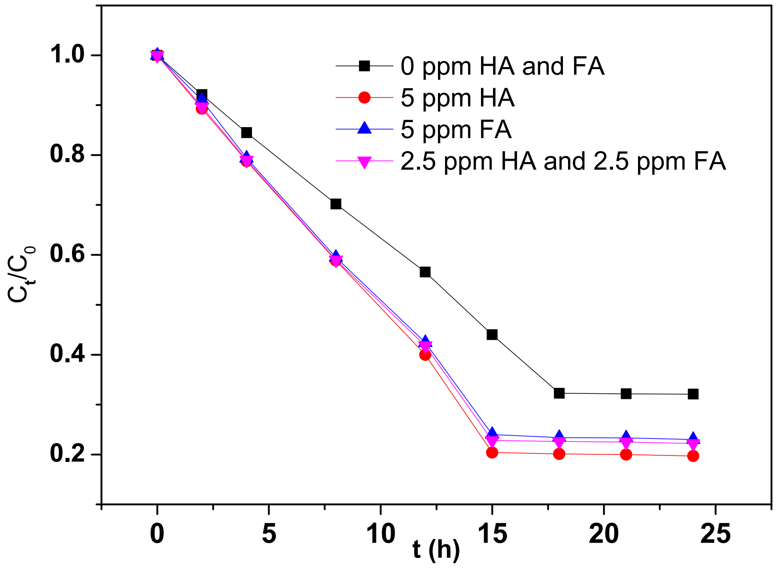

Figure S3. TC sorption behavior on magnetic sludge biochar as a function of the co-existence of HA and FA.
